# Electrical impedance tomography in calves with bovine respiratory disease: correlations with clinical and blood gas findings

**DOI:** 10.3389/fvets.2025.1556943

**Published:** 2025-05-01

**Authors:** Ulrich Bleul, Fabienne Kluser, Andreas Waldmann, Christian Gerspach

**Affiliations:** ^1^Large Animal Reproduction, Clinic of Reproductive Medicine, Department of Farm Animals, Vetsuisse-Faculty University Zurich, Zurich, Switzerland; ^2^Clinic for Ruminants, Department of Farm Animals, Vetsuisse-Faculty University Zurich, Zurich, Switzerland

**Keywords:** electrical impedance tomography, pneumonia, bovine, respiratory disease, thorax, ventilation

## Abstract

Bovine respiratory disease (BRD) is a multifactorial global problem associated with long-term deleterious effects on the well-being of calves and marked financial losses. Prompt diagnosis of BRD, monitoring the success of treatment, and providing an accurate prognosis remain challenging because current methods for stall-side diagnosis are inadequate. To improve diagnosis in addition to clinical and morphological findings and gain insight into the respiratory dynamics of BRD, thoracic electrical impedance tomography (EIT) was used to evaluate calves with BRD (Group D; *n* = 42) and healthy calves (Group H; *n* = 13). Thoracic EIT is a non-invasive method of quantifying differences in impedance changes between various lung regions and impedance changes over time. A belt with 32 equidistantly mounted electrodes was placed around the thorax of non-sedated calves of both groups to measure impedance changes during respiration. The results were compared with the clinical findings and the California BRD scores. Compared with group H, Group D had decreased ventilation in the ventral lung regions (*p* = 0.05); ventilation shifted to the left lung lobes in calves with marked auscultatory changes (*p* = 0.013). In addition, the quartile ventilation ratio on inspiration (V_QRi_), used to quantify changes in impedance during inspiration, differed significantly between the two groups (*p* = 0.0039). Of all the EIT parameters, V_QRi_ correlated most closely with paO_2_ and the A-a-gradient and was significantly lower in group D than in group H (*p* = 0.061). The results of EIT revealed differences in the inspiratory dynamics of clinically healthy and ill calves and correlated with the clinical and blood gas findings. Thus, EIT can be used alone or together with other diagnostic tools to identify and monitor BRD in calves.

## 1 Introduction

Bovine respiratory disease (BRD) may be the most important disease complex affecting replacement and veal calves. In the United States, BRD is the second most common health problem in dairy calves before weaning with an incidence of 18% after intestinal diseases and the most common health problem after weaning with 11.2%. Not only does BRD affect the well-being of calves, but it is associated with the use of large quantities of antibiotics and marked economic losses (1, 2). Bovine respiratory disease is also known as infectious bronchopneumonia, shipping fever, or undifferentiated fever (3, 4). Calves are most susceptible to BRD in the first months of life when maternal immunity wanes and stressors such as transport and grouping occur concurrently (5, 6). A uniform definition of this complex has not been established because the development of disease is affected by various environmental factors; the health status of the calf, and the response of the calf to infection with different pathogens. In addition, differentiating BRD from other respiratory diseases can be challenging (7, 8). The most common clinical signs include: an abnormal general demeanor, pyrexia, decreased appetite, nasal discharge, cough, dyspnea, tachypnea, and increased lacrimation (8). However, the correlation between clinical signs and postmortem findings is only moderate (9). For this reason, studies have evaluated numerous methods for diagnosis of bronchopneumonia and its severity, including continuous monitoring of body temperature, demeanor, and appetite, and auscultation, radiography, ultrasonography, and computed tomography (CT) of the thorax (7). However, these diagnostic methods only reveal changes in the morphology of the respiratory tract. Although these diagnostic methods agree relatively well with the pathologic findings (10), they do not provide information about the severity of lung function impairment; dynamic function tests are required for this. Various spirometric methods have been used to determine changes in respiration attributable to pathologic processes or treatment regimens (11–15). Electrical impedance tomography (EIT) has been increasingly used in veterinary medicine to determine lung ventilation distribution in anesthetized and non-anesthetized animals of various species (16–20). This non-invasive, radiation-free, imaging modality measures thoracic impedance changes in real-time (17). Un-detectable alternating current is applied through pairs of electrodes in a belt attached to the thorax of the animal. The intrathoracic tissue allows the passage of the electrical currents with little resistance, but air in the lungs increases resistance. The resulting change in electrical impedance is measured by the remaining electrodes creating a dynamic image of the respiratory cycle. Electrical impedance tomography has been used to describe changes in lung ventilation in newborn calves in the postnatal period (21), and in a more recent pilot study, various parameters were used to visualize changes in the respiratory dynamics in cows with pneumonia (22).

The goal of this case–control study was to describe and compare ventilation in calves with clinical signs of bronchopneumonia and clinically healthy calves using EIT. A diagnosis of bronchopneumonia was based on the results of clinical examination that included characteristic auscultatory changes and an abnormal demeanor. We hypothesized that the data generated by EIT differ between clinically healthy and ill calves and that the changes correlate with those of blood gas analysis.

## 2 Materials and methods

A total of 42 diseased (group D) and 13 healthy calves (group H) were enrolled in the study. Group D included calves that were admitted to our clinic as patients because of an individual illness or because of a herd problem, and the healthy calves originated from the university-owned research herd or from a private herd. Diseased calves had nasal and ocular discharge, dyspnea or tachypnea, abnormal lung sounds, and a decreased appetite. Healthy calves were only used in the study if they did not have any of these signs. Written consent to participate in the study was available for all calves not owned by the university.

The age of the calves on admission ranged from 6 to 379 days (median ± SE, 60.0 ± 9.20 days). There were 24 bull calves and 31 heifers, weighing between 35 and 200 kg (x̅ = 98 ± 37 kg). Breeds included Brown Swiss (n = 21), mixed-breed (n = 16), Holstein Friesian (n = 6), Simmental (n = 4), Limousin (n = 3), Swiss Braunvieh (n = 2), Angus (n = 1), Montbéliard (n = 1), and Red Holstein (n = 1).

The calves were kept in straw-bedded pens with free access to hay and water. Calves received milk replacer two to six times a day from a bottle or a bucket in an amount that was adjusted to their weight and to the amount of solid feed consumed.

A history of the clinical signs and their duration was obtained on admission. The illness was considered peracute to acute when the duration was less than 2 days, subacute to chronic when the duration was 3 days to 4 weeks, and chronic when it had persisted longer than 4 weeks.

On admission and every day during hospitalization, the calves underwent a clinical examination. Calves were also assessed using a BRD scoring system (23). For the latter, ocular discharge and droopy ears, nasal discharge, coughing, and rectal temperature were assessed and assigned a score of 0 (normal), 1 (slightly abnormal), 2 (abnormal) and 3 (severely abnormal). The total score was 12 in calves in which all the signs were severely abnormal and 0 in healthy calves (24).

During auscultation of the lungs, both sides of the thorax were examined in horizontal lines from dorso-cranial to ventro-caudal. Abnormal lung sounds were categorized into 3 groups and scored (1, inconspicuous or mild; 2, moderate; 3, severe) (25). Abnormal lung sounds included increased bronchial sounds, crackles, wheezes, pleural friction rubs, and the absence of breath sounds (26).

Based on the results of the clinical examination, the calves were treated according to the standards of the University of Zurich Veterinary Hospital. Treatment followed the guidelines of the Vetsuisse Faculty in cooperation with the Swiss Veterinary Association and the Federal Office for Food Safety and Veterinary Affairs (27); at no time did aspects of the study affect treatment.

Arterial blood gas analysis was carried out at the time of admission as well as at discharge. Arterial blood was collected from the medial ramus intermedius artery of the caudal auricular artery (28). The hair over the collection site was clipped, and the area was disinfected with 70% ethyl alcohol and anesthetized topically with sterile 5% prilocaine/lidocaine cream (Emla cream, Aspen, Dublin, Ireland). A 24G hypodermic needle was inserted into the artery and the blood was collected into a heparin capillary tube (B. Braun, Melsungen, Germany) and immediately analyzed (RapidPoint 500, Siemens Healthcare Diagnostics, Camberley, United Kingdom). The following variables were analyzed or calculated from analyzed variables: pH, paCO_2_, paO_2_, HCO_3_^−^, base excess, hematocrit, oxygen saturation, anion gap, and concentrations of glucose and lactate.

For the calculation of the alveolar-arterial oxygen difference (A-a gradient), the alveolar partial pressure of oxygen pAO_2_ was first calculated as follows (29):


$${paO}_{2}={FiO}_{2}\;\left(\mathrm{pAtm}-{pH}_{2}O\right)-\left({\mathrm{paCO}}_{2}/RQ\right)$$

where FiO_2_ is the inspired fraction of oxygen from natural air set at 0.21, pAtm is the prevailing barometric pressure (mmHg), pH_2_O is partial water pressure (47 mmHg), paCO_2_ is the arterial partial pressure of carbon dioxide (mmHg), and RQ is the respiratory quotient (1).


$$A-a\;\mathrm{gradient}={pAO}_{2}-{paO}_{2}$$

where pAO_2_ is the alveolar partial pressure of oxygen (mmHg), and paO_2_ is the arterial partial pressure of oxygen (mmHg).

**Figure 1:**
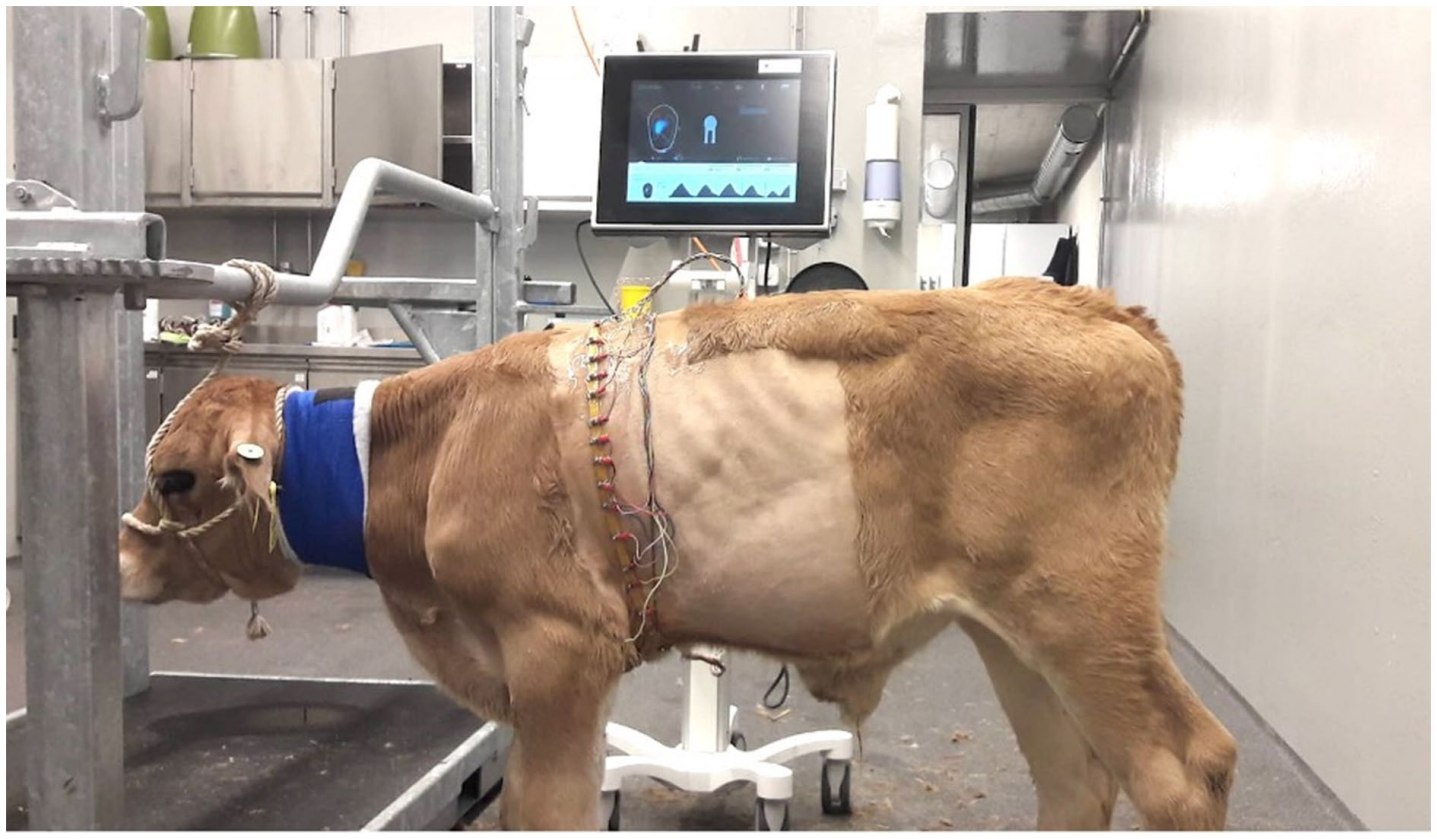
EIT measurement in a calf with respiratory disease (group D). The rubber belt is at the level of the 6th intercostal space and is equipped with 32 evenly spaced electrodes.

Each calf underwent EIT at the time of entry and discharge from the study using a Swisstom BB^2^ device (SenTec, Landquart, Switzerland). Asingle plain, elastic belt equipped with 32 electrodes was attached to the thorax behind the elbows in such a way that it was in contact with the 6^th^ intercostal space midway between the sternum and vertebral column of the calf (Figure 1). The belts used were home-made from elastic rubber bands in three different sizes (58.5 cm, 75.5 cm or 100 cm in the non-stretched state) depending on the calf’s chest girth. The Swisscom BB^2^ device was connected to the belt mounted with electrodes. To match the results of the EIT measurements with the anatomically correct regions of the lungs, the finite element model, described in another study (21), was used. Briefly, image segmentation of the contours of the lungs, heart, and thorax at the level of the 6^th^ intercostal space was done using CT scans of 6 calves; the finite element model generated allowed the depiction of the EIT results of the lungs.

Three belt sizes were used depending on the size of the calf; they measured 58.5, 75.5, and 100 cm in a non-stretched state. The intermediate size was used most frequently. The area of the 6^th^ intercostal space was clipped, and contact gel (Vetogel, Streuli, Uznach, Switzerland) was applied for better contact between the electrodes and the skin. The belt was applied 10 min before the measurements started to accustom the calf to it. A current of 3 mA and a frame rate of 50 *frames* per second were used. Measurements were conducted over a minimum of 21 breaths. The recorded data were analyzed retrospectively using ibeX software (SenTec, Landquart, Switzerland) and transferred to an Excel data sheet or PDF.

### 2.1 Respiration rate and inspiration time

The determination of respiratory rate was based on the number of terminations of inspirations that were identified using EIT. In addition, the difference between the start and the end of individual inspiration cycles was determined and reported as inspiration time (in seconds).

### 2.2 Global expiratory tidal impedance variation

TIV_EXP_ was calculated from the change in impedance of the entire lung between the start and end of expiration and was reported in arbitrary units. Based on an existing linear relationship, TIV_EXP_ can be used as a proxy variable for spirometrically determined tidal volumes (17).

### 2.3 Quartile ventilation ratio during inspiration or expiration

**Figure 2:**
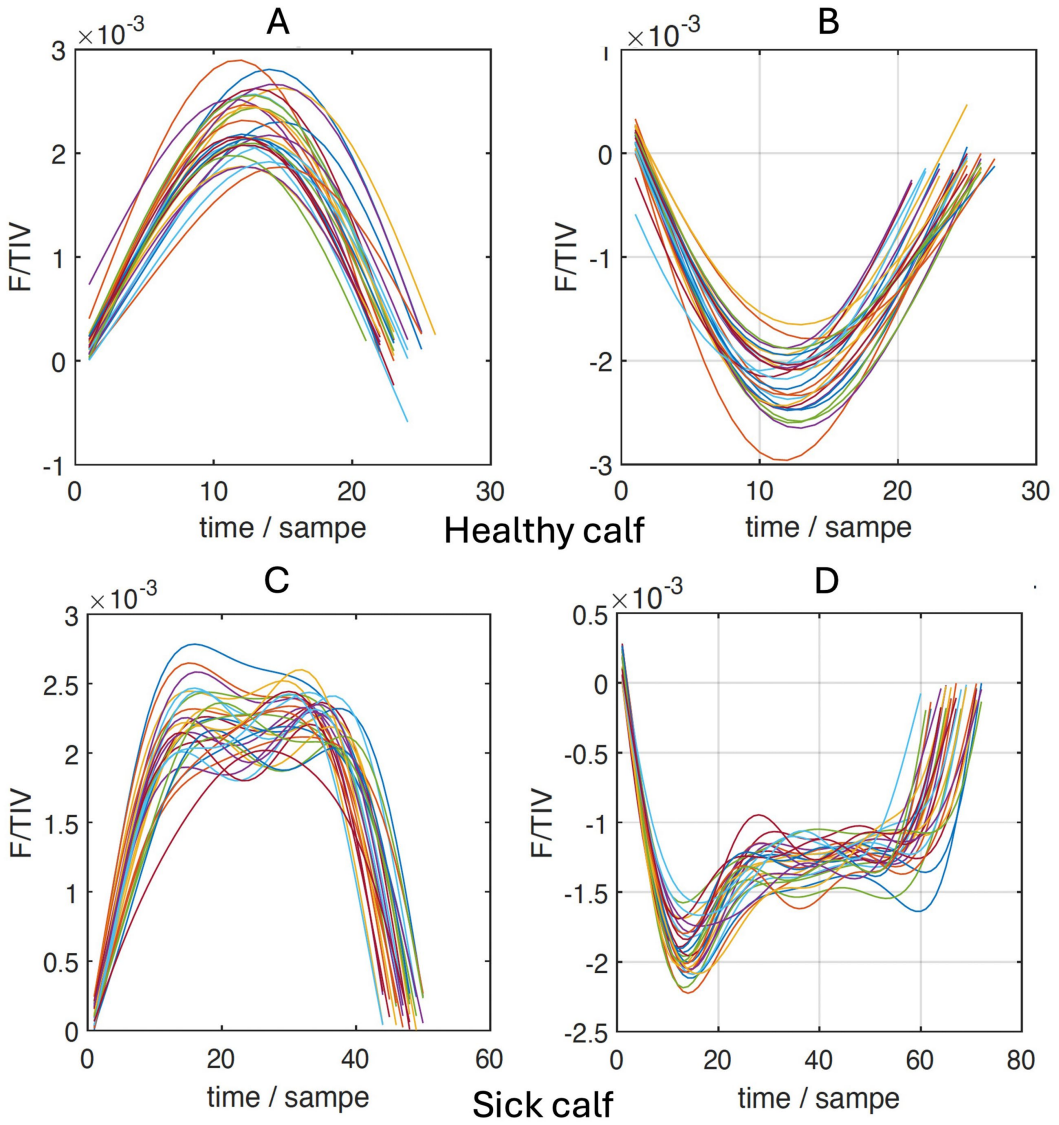
Sections of inhalation **(A,C)** and expiration **(B,D)** of the flow/tidal impedance variation (F/TIV) curves over time of a healthy **(A,B)** and a sick calf **(C,D)**.

To describe the impedance and its changes over the time of a breath, the parameter F/TIV was used, and a curve was generated from its values. TIV refers to the change in impedance over time in a single breath and is used as a substitute for volume. The quotient of the flow (F), which is the first derivative of TIV, and TIV is then plotted over time (Figure 2). Alterations in the curve can be used to quantify an inhomogeneity in the lung filling and thus a disturbed lung compliance. To calculate mathematical deviations from an even semi-circular flow curve (the upper 2 graphs in Figure 2) during inspiration and expiration, the curve was divided into quartiles (Q1-Q4), and the ratio of the sums of Q1 and Q4 to the sums of Q2 and Q3 was calculated (22):


$$V_{QR}=\frac{\left(Q1+Q4\right)}{\left(Q2+Q3\right)}$$

### 2.4 End-expiratory lung impedance and end-inspiratory lung impedance

These two variables approximately describe the lung volume at the end of expiration (EELI) and at the end of inspiration (EILI) and are reported in arbitrary units. They can be used to show a change in lung volume between different points in time or treatment methods (30).

### 2.5 Center of ventilation

The measurement of electrical impedance allows the creation of a two-dimensional depiction of the laterolateral and ventrodorsal distributions of the ventilation of both lungs. The *center of ventilation (CoV)* has been defined as the weighted mean of the geometric centers of ventilation and is reported as a percentage of the thoracic cross-section (31, 32). When the ventrodorsal (CoV_VD_) or the right–left (CoV_RL_) distribution is 50%, the distribution of ventilation is even and the CoV is at the center. Higher values indicate a shift in ventilation dorsally or to the left. Accordingly, CoV_VD_ or CoV_RL_ with a 100% value describe ventilations that occur in the dorsal most and left most regions of the lungs (17).

### 2.6 Dependent silent space or non-dependent silent space

Regions of the lungs with impedance changes that were smaller than 10% of the maximum changes of the entire lung were defined as so-called silent spaces. It can be assumed that silent spaces are characterized by no ventilation or hypoventilation. The calves were always examined in a standing position or in sternal recumbency. Therefore, silent spaces dorsal to the horizontal line at the level of the CoV were calculated as non-dependent silent spaces (NSS), which were not gravity-dependent, and silent spaces ventral to this line as (gravity) dependent silent spaces (NSS) (33). Silent spaces were recorded in percent (21).

### 2.7 Ventilated area (A)

The ventilated area (A) describes the dorsal and ventral and the left and right portions, respectively, of the ventilated regions relative to the entire impedance changes of the lungs, reported in percent.

### 2.8 Data analysis and availability

The program StatEL Program (AdScience, Paris, France) in Microsoft Excel (Microsoft Windows, Wallisellen, Switzerland) was used for statistical calculations. Means and standard deviations were calculated for continuous variables. Dependent and independent variables with normal distribution were analyzed using the t test. Dependent variables with non-normal distribution were analyzed using the Wilcoxon test and independent variables with non-normal distribution with the Mann–Whitney test. Differences among multiple groups were analyzed using the Kruskal-Wallis test because the requirements for ANOVA were not met. Linear relationships between variables were tested using the Spearman test. A *p*-value <0.05 was considered significant.

All data are available from the corresponding author on reasonable request.

## 3 Results

### 3.1 Clinical examination

The age of the calves in groups D (72.5 ± 9.11 days) and H (53.0 ± 26.15 days) (both median ± SE; *p* = 0.15), the distribution between the two sexes (*p* = 0.4), and the weight of the calves (94.64 ± 37.30 kg vs. 108.4 ± 35.69 kg; *p* = 0.25) did not differ. The illness was acute in 18 calves, subacute in 21, and chronic in 3.

The two groups of calves did not differ with respect to rectal temperature on admission (39.34 ± 0.61°C vs. 39.02 ± 0.77°C; *p* = 0.14) but calves of group D had significantly higher California BRD scores (7.05 ± 2.11 vs. 3.69 ± 1.38; *p* < 0.00001). Likewise, the ocular and nasal discharge scores also differed significantly between diseased and healthy calves (1.26 ± 0.67 vs. 0.31 ± 0.28; *p* = 0.00019).

After the initial examination and the first EIT measurement or in the subsequent days, 11 of the 42 calves were euthanized because of a hopeless prognosis.

### 3.2 Blood gas variables

The paO_2_ on admission was significantly lower in diseased calves than in healthy calves (70.37 ± 18.95 vs. 81.12 ± 9.6 mmHg, *p* = 0.017), whereas the A-a gradient was significantly higher in the diseased calves (32.27 ± 11.45 vs.15.42 ± 8.03 mmHg, *p* < 0.013). In the calves of group D that were discharged, the A-a gradient decreased significantly during the study from 29.54 ± 16.0 mmHg on admission to 20.25 ± 9.30 mmHg at discharge (*p* = 0.038) and no longer differed from group H. The oxygen saturation differed significantly between sick and healthy calves on admission (92.40 ± 7.92% vs. 96.05 ± 1.67%, *p* = 0.045) as well as at discharge (95.64 ± 2.83% vs. 97.80 ± 1.48%, *p* = 0.02).

### 3.3 Electrical impedance tomography

#### 3.3.1 Examination at admission

**Figure 3:**
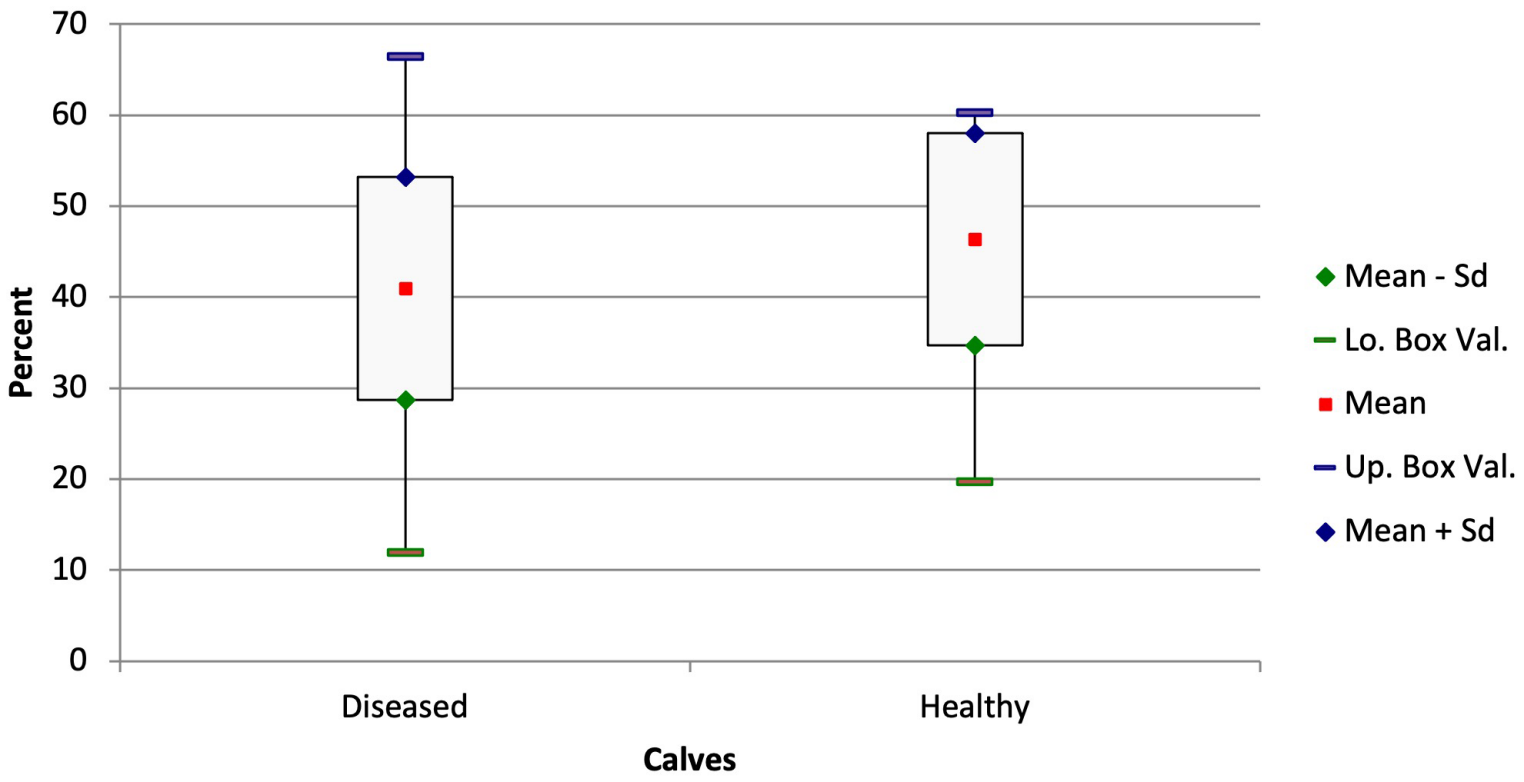
Box and whisker plots of the ventilated areas in the ventral thorax in diseased and healthy calves (*p* = 0.05).

The diseased calves had a significantly smaller portion of ventilated lung tissue (A) in the ventral portion of the lungs compared with the healthy calves (ventral ventilated area: 40.99 ± 12.26% vs. 46.35 ± 11.67%; Figure 3; *p* = 0.05). A similar disparity in the distribution of ventilated areas was also found when comparing the subacute and chronically ill calves with the healthy calves (ventral ventilated area: 39.51 ± 11.67% vs. 46.35 ± 11.67%; *p* = 0.019) but not in the calves with a shorter duration of illness and the healthy calves (*p* = 0.51).

**Figure 4:**
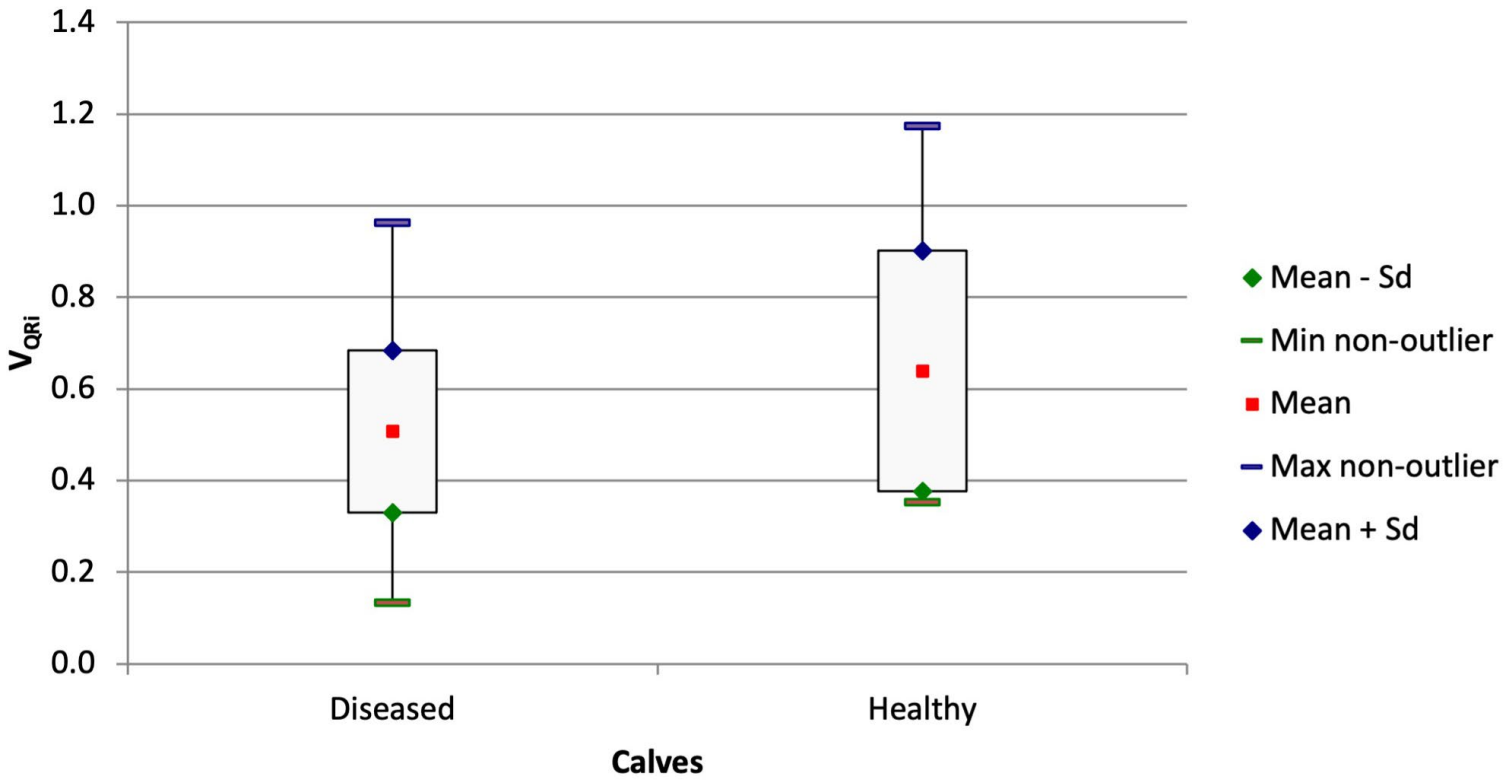
Box and whisker plots of the V_QRi_ at admission in calves with respiratory disease and in healthy calves (*p* = 0.044).

The quartile ventilation ratio during inhalation (V_QRi_) also differed between the groups (Figure 4; *p* = 0.044). The F/TIV curve of the diseased calves had a different shape and resulted in a lower V_QRi_ compared with the healthy calves (0.51 ± 0.18 vs. 0.64 ± 0.26; *p* = 0.044). Similar to the ventilated area in the ventral portion of the lungs, the difference between the chronically ill and healthy calves was significant (0.48 ± 0.64 vs. 0.64 ± 0.26; *p* = 0.047) but not the difference between the ill calves with a shorter duration of illness and the healthy calves (*p* = 0.17).

Calves with moderate or severe auscultatory findings had a shift of ventilation to the left compared with calves with only mild or no lung sounds at all. Calves with moderate or severe lung sounds had a CoV_RL_ of 54.41 ± 7.41% and those with no or only minimal lung sounds had a CoV_RL_ of 48.64 ± 6.91% (*p* = 0.013). The ventilated area of the left lung was also greater in the calves with moderate or severe lung sounds (49.09 ± 14.10%) compared with calves with mild lung sounds (37.98 ± 12.85%, *p* = 0.012).

**Figure 5:**
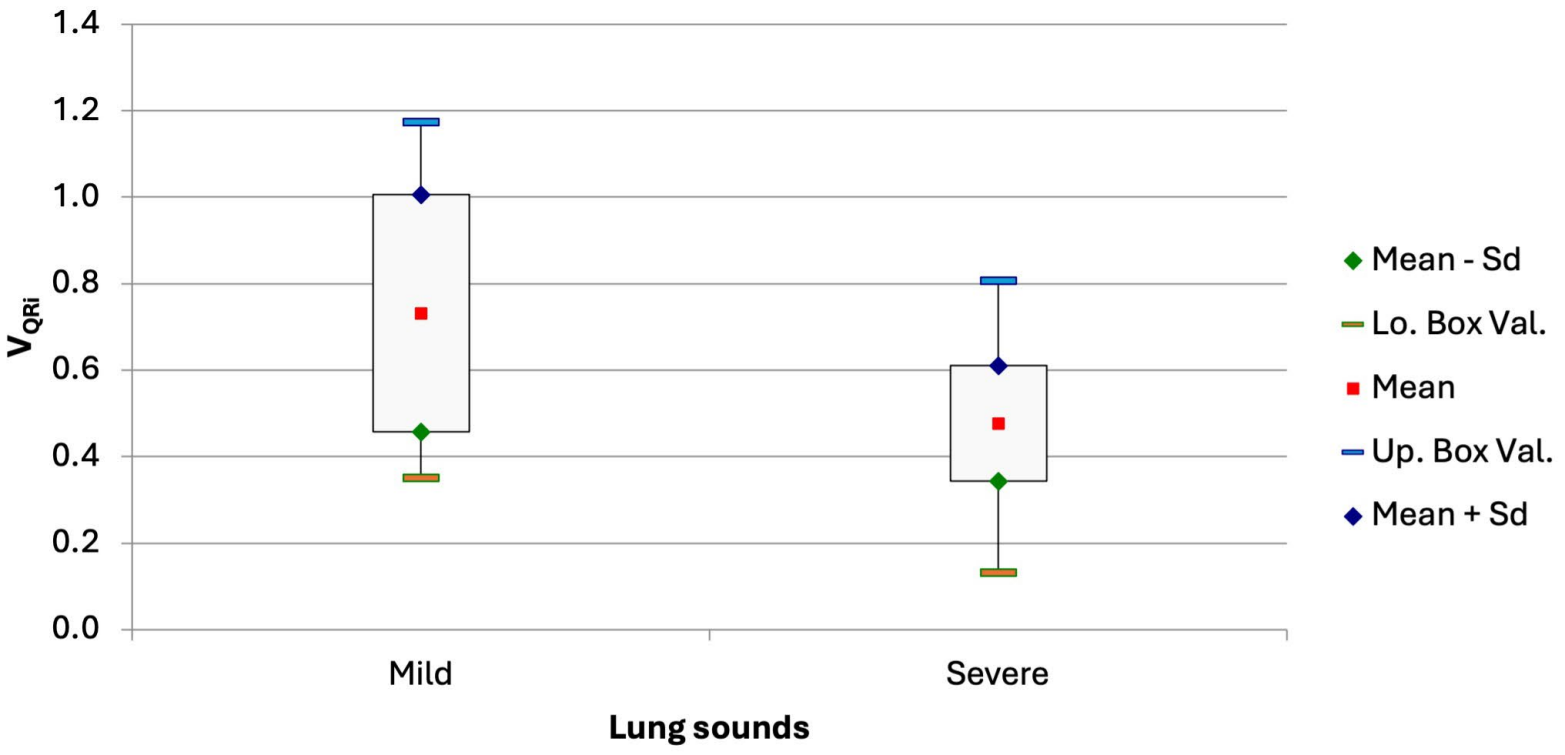
Box and whisker plot of the V_QRi_ in calves with no or only mild lung sounds and in calves with moderate or severe lung sounds (*p* = 0.0039).

The V_QRi_ was significantly lower in calves with pronounced lung sounds than in calves with mild or no lung sounds (0.48 ± 0.13 vs. 0.73 ± 0.27; Figure 5; *p* = 0.0039). This variable had a modest correlation with the California BRD score (V_QRi_ = −0.02276*score + 0.6817; r = −0.27, *p* = 0.043).

The paO_2_ correlated with several EIT variables, which are listed in Table 1. Furthermore, the variables RR, CoV_vd_, and DSS correlated positively and the variables NSS and V_QRi_ negatively with the A-a gradient.

**Table 1:** Correlations between EIT variables.

	*paO_2_*			A-a gradient		
Variables	Correlation equation	r	*p*-value	Correlation equation	r	*p*-value
RR	RR = −0.3488*pO2 + 61.87	−0.32	0.017	RR = 0.5273* A-a gradient + 20.81	0.414	0.0016
CoV_RL_	CoV_RL_ = −0.1246*pO2 + 62.28	−0.289	0.03		0.231	0.099
CoV_VD_		−0.259	0.053	CoV_VD_ = 0.09951* A-a gradient+ 53.1	0.288	0.037
NSS		0.203	0.13	NSS = −0.1199*A-a gradient + 12.57	−0.338	0.013
DSS	DSS = −0.1717*pO2 + 30.65	−0.300	0.023	DSS = 0.1954* A-a gradient + 12.46	0.296	0.032
V_QRi_	V_QRi_ = 0.004845*pO2 + 0.18	0.424	0.00083	V_QRi_ = −0.005935*A-a gradient + 0.7124	−0.465	0.00032

RR, respiratory rate; CoV, center of ventilation; DSS, dependent silent space; NSS, non-dependent silent space; V_QRi_, quartile ventilation ratio during inspiration; and the pO_2_ in arterial blood (paO_2_) and the A-a gradient at admission (correlation coefficient, r).

#### 3.3.2 EIT findings before and after treatment

**Figure 6:**
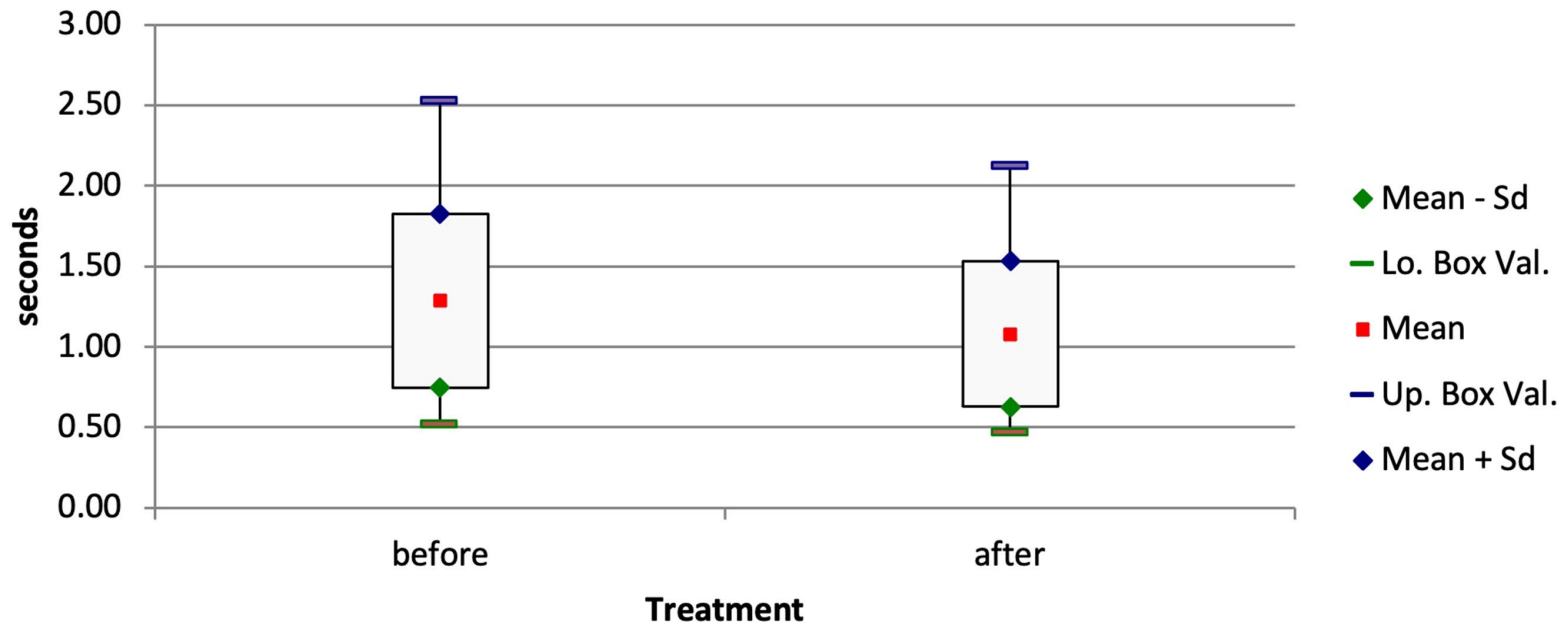
Box and whisker plots of the inspiration time of surviving calves with respiratory disease before and after treatment (*p* = 0.04).

In calves of group D that were treated and discharged, the mean EELI and EILI values decreased by 12% between the EIT measurements at admission and those at discharge (EELI, *p* = 0.015; EILI, *p* = 0.016). Likewise, the inspiration time in these calves decreased significantly from 1.28 ± 0.54 s to 1.08 ± 0.45 s (Figure 6; *p* = 0.04).

#### 3.3.3 Comparison of EIT results of surviving and non-surviving calves

The CoV_RL_ of calves with respiratory disease that were euthanized because of a hopeless prognosis was significantly higher than in calves that were discharged (57.15 ± 5.9% vs. 52.02 ± 7.72%, *p* = 0.045). Comparison of the ventilated areas of the right and left lungs gave analogous results; in the euthanized calves, 44.99 ± 11.95% of the right lung was ventilated compared with 55.67 ± 14.41% (*p* = 0.028) in the discharged calves. Furthermore, there was a trend for a lower V_QRi_ in non-surviving calves compared with surviving calves (0.439 ± 0.124 vs. 0.562 ± 0.215, *p* = 0.061).

## 4 Discussion

Bovine respiratory disease is a disease complex that primarily affects calves in the first six months of life. Young calves are at increased risk because of stress associated with transport and pooling of animals from different sources (34). Preventive antibiotic treatment is therefore commonly used in young calves to minimize losses caused by BRD. Not surprisingly, respiratory disease in calves is by far the largest reason for antibiotic use among all farm animals in Switzerland (35). Typically, antibiotic treatment is given prophylactically regardless of medical indication because co-mixed calves are often at different stages of BRD; calves may be healthy or have viral tracheitis, bronchitis, or severe purulent bronchopneumonia (34).

For effective and targeted treatment of calves with respiratory disease, one should be familiar with the different stages of the illness and be able to differentiate healthy and sick calves. A standardized clinical examination is the best way to achieve this because it allows better comparison of clinical findings among calves in a group or from different sources (3). For this purpose, we used the California BRD scoring system, a commonly used method of assessing BRD in calves, which yielded significantly different scores for diseased and healthy calves. However, the sensitivity and specificity of these scoring systems are only moderate (7). Auscultation of the thorax confirmed the differences between the healthy and diseased calves but similar to scoring systems, auscultation has a much lower sensitivity than ultrasonography for the detection of pathologic lung changes (36). In addition to the need for an experienced examiner, ultrasonography also has diagnostic limitations: Functional changes cannot be detected and only the morphologic changes near the surface of the lungs can be visualized (34). In contrast, EIT allows the visualization of dynamic lung changes, which are more likely to correspond to the auscultatory findings.

Several EIT variables differed significantly between calves with moderate or severe lung sounds and healthy calves including the CoV_RL_, which shifted to the left in diseased calves. Similar to humans and horses, ventilation in calves is not evenly distributed between the right and left lungs because of anatomic factors. In the first month of life, about two-thirds of the inhaled air enters the right lung (21). In calves with no or only mildly abnormal lung sounds, the CoV_RL_ was to the right of the theoretical center of all ventilated lung regions (48.64%) as expected. However, in calves with severe lung sounds the CoV_RL_ was to the left of the theoretical center. The most likely reason for this was increased filling of the left lung with air or a decreased filling of the right lung. The inflammation that is associated with bronchopneumonia spreads along the airways and therefore alveolar atelectasis and congestion, which are typical for cattle, mostly affect cranial regions of the lungs (37). Thus, the right lung lobe, which has a direct bronchial connection to the trachea, is affected earlier and more severely in bronchopneumonia than the left lung (34); this leads to a decrease in the ventilated area of the right lung. The 11 calves that were euthanized after the initial examination because of a hopeless prognosis had a CoV_RL_ that was even further to the left (57.15%) than that of the discharged calves (52.02 ± 7.72). This value was significantly lower than that of the euthanized calves but not in the range of a normal ventilation distribution (<50%). Whether the CoV_RL_ shifted back to the right side after discharge from the hospital because of improvement in ventilation is not known; however, the variable CoV_RL_ may be helpful in treatment decisions.

Based on the anatomic characteristics of the bovine lungs, the cranial parts, particularly on the right side, are primarily affected in calves with BRD and the ventral parts have the most pronounced macroscopic lesions in chronic cases (38, 39). This involves bronchial and alveolar damage that can lead to the collapse of these regions, with subsequent consolidation and necrosis. These abnormalities can be detected using EIT. For instance, ventilation of the ventral lung regions was reduced by more than 5% in diseased calves. However, only calves with subacute or chronic illness had a reduction in ventilated lung areas, while those with acute illness did not. It can be assumed that the latter had acute interstitial pneumonia (38) with sufficient compliance of these parts of the lungs.

The difference in the V_QR_, which was recently described in cattle by Brabant and co-workers (22), also suggests that lung compliance in calves with respiratory disease is reduced compared with healthy calves. The flow/tidal impedance variation curves (Figure 2), which can be quantified using V_QR_, show an inhalation pattern in diseased calves that differs from that of healthy calves. The means of V_QRi_ were lower in the sick calves, which can be interpreted as a decrease in the elasticity of the lungs. Similar to the measurements of the ventilated regions of the ventral lung, the difference in V_QRi_ between the diseased and healthy calves was attributable to the chronically ill calves because their V_QRi_ was significantly lower than that of acutely ill calves. Therefore, the variable V_QRi_ may also be a sign of inhomogeneity in lung filling (22). This finding is in agreement with clinical observations; V_QRi_ was negatively correlated with the California BRD score and was lower in calves with severe lung sounds. It can be assumed that in calves with bronchopneumonia, lung sounds increase with increasing severity in parallel with a decrease in compliance. However, lung auscultation as well as the California BRD score have a relatively low sensitivity for the detection of lung consolidation (36), and this could explain why these two variables were only poorly correlated with V_QRi_.

The inspiration time also suggests that bronchopneumonia caused a reduction in lung compliance in the diseased calves; the inspiration time decreased after treatment and was shorter than at the time of admission to the clinic. An increased inspiration time indicates inspiration dyspnea, as this occurs mainly as a consequence of decreased expansivity of the lungs (34). In contrast, an expiration time that exceeds the inspiration time indicates peripheral bronchoconstriction; however, this and changes in V_QRe_ were not observed in the present study. Likewise, in a study of adult cattle with respiratory disease, changes in V_QRe_ were not observed (22). This variable can also indicate stenosis of the airways such as bronchospasm. It is not clear whether this did not occur in the calves of our study or whether it was missed by EIT because of its lens-shaped measuring range, in which the cranial parts of the airways are not detected.

Both the EILI and EELI decreased from the first EIT to the second after treatment. This means that the maximum (EILI) and minimum impedance (EELI) as well as lung expansion decreased during one breath (40). The difference between EILI and EELI corresponds to the tidal variation and is an approximate description of the spirometrically determined tidal volume (41). Based on the similar decrease in the two variables in our study, the tidal volume does not appear to have been affected by treatment. The EELI alone can also serve as an approximation of air-filled lung (42). This suggests that the calves had a smaller lung volume after treatment than before, although this is counterintuitive. Another explanation is that the diseased calves were dehydrated on admission, and rehydration during treatment, which in some cases included intravenous infusion of fluids, restored normal hydration. The increased blood and fluid volume may have led to an increase in lung conductivity and thus to a decrease in impedance (43).

Highly significant differences were seen in the paO_2_ and A-a gradient in the diseased and healthy calves at the time of admission. Some studies reported similar results (44, 45) while others did not (46, 47). Of note, clinically healthy calves in the present study had mean paO_2_ and A-a gradient values that differed considerably from those of other studies. For example, the mean paO_2_ of the healthy calves was 81 mmHg, which was at the lower reference interval limit used by Ellis et al. (45), and the mean A-a gradient of 15 mmHg was higher than their reference interval of <10 mmHg (48, 49). Clear differentiation of diseased and healthy calves seems therefore not always possible because of the low sensitivity of the clinical tests used (36, 46, 50). Thus, when correlations between any of those two variables and an EIT variable were found, we used all the calves from both groups for calculations.

The variables respiratory rate, CoV_RL,_ and DSS were negatively correlated with paO_2_. It is known that a decrease in blood oxygen levels increases the respiratory rate and the respiratory rate measured by impulse oscillometry is even more closely correlated with the paO_2_ than in our study using EIT (34, 51).Bronchopneumonia decreases the tidal volume resulting in a compensatory increase in the respiratory rate. This is of particular significance in ruminants because unlike humans and other animal species, they are unable to ventilate alveolar regions via collateral airways when bronchial obstruction occurs (52). As discussed, the shift in CoV_RL_ toward the left lung and CoV_VD_ toward the dorsal lung regions and an increase in DSS corresponds to the typical spread of bronchopneumonia in cattle. Bronchopneumonia starts in the ventral and right regions of the lungs. At some point, inadequate ventilation cannot be compensated, leading to a decrease in paO_2_ and an increase in the A-a gradient (37, 53). However, the correlations between CoV_RL_, CoV_VD_, and DSS and the blood gas variables paO_2_ and the A-a gradient were only moderate. The extent of lung tissue affected cannot be estimated using paO_2_ and the A-a gradient. Published information concerning the usefulness of paO_2_ to quantify the extent of lung damage is contradictory (45, 46). The variable V_QRi_ may allow a better estimation of the extent of lung damage because it was most closely correlated with paO_2_. As the ability to absorb oxygen decreases, the quartile ventilation ratio changes because of changes in impedance and impedance differences during inspiration. In contrast to healthy calves, the changes in the inspiration pattern of diseased calves were primarily characterized by a smaller and uneven increase in impedance in mid-inspiration (Figure 2). Thus, calves with bronchopneumonia seem to have reduced and/or inhomogeneous compliance of the lungs (22), which increases with increasing lung damage, as does the ability to absorb oxygen. This is also supported by the negative correlation between the A-a gradient and the V_QRi_; of all EIT variables, V_ORi_ had the closest correlation with the A-a gradient. A discrepancy between ventilation and perfusion of the lungs (V/Q mismatch), which may result from pneumonia, can be quantified using the A-a gradient. Inflammatory lung changes result in decreased ventilation in the affected alveoli, which results in redistribution of blood flow away from the collapsed or severely inflamed lung regions (54). A recent sonographic study that investigated the extent of lung damage showed that the A-a gradient was useful in differentiating calves with and without bronchopneumonia (46). Therefore, it should be possible to use the V_QRi_ for the same purpose provided that the problem of quantification of the affected lung regions can be resolved in future studies. There are limitations of the study: Besides a relatively small sample size the classification of calves based solely on clinical findings, which does not allow the assessment of the severity of bronchopneumonia (55, 56). This would necessitate computed tomographic or pathologic evaluation, which is expensive, invasive, and less suitable for large studies. Furthermore, these are not cow-side tests. Thoracic radiography and ultrasonography generate more accurate diagnoses than clinical examination and scoring systems. Ultrasonography can be easily done in large groups of cattle (57), but thoracic radiography is more time-consuming and it is technically more challenging to visualize the entire lung field in larger calves. In combination with these methods, EIT may be an important tool for the diagnosis of changes in morphology and respiratory dynamics in calves with BRD. For this purpose, it would be useful to obtain information on the differences between acutely and chronically ill calves and to demonstrate the usefulness of EIT in the field.Conclusion

The present study used electrical impedance tomography (EIT) to determine dynamic respiratory changes associated with bronchopneumonia in calves. The changes in impedance during a breathing cycle and the differences in impedance corresponded to findings of pathologic and spirometric studies in calves with bronchopneumonia. Diseased calves had decreased ventilation in the ventral and cranial lung regions, and an increase in non-ventilated areas in the ventral lung regions was associated with deterioration in oxygenation. The quartile ventilation ratio during inspiration (V_QRi_) appears to be the most suitable parameter for recognising changes in the breathing pattern. Further studies are required to determine the extent these dynamic changes correspond with morphologic changes and how EIT can improve the diagnosis and prognosis and aid in monitoring response to treatment.

## Data Availability

The original contributions presented in the study are included in the article/supplementary material, further inquiries can be directed to the corresponding author.
